# Clinical characteristics of definite vestibular migraine diagnosed according to criteria jointly formulated by the Bárány Society and the International Headache Society

**DOI:** 10.1016/j.bjorl.2021.12.004

**Published:** 2022-01-21

**Authors:** Zhe-Yuan Li, Bo Shen, Li-Hong Si, Xia Ling, Kang-Zhi Li, Xu Yang

**Affiliations:** a Department of Neurology, Aerospace Center Hospital, Peking University Aerospace School of Clinical Medicine, PR China; b Department of Neurology, The First Affiliated Hospital of Jinzhou Medical University, PR China

**Keywords:** Vestibular migraine, Vestibule, Migraine, Videonystagmography, Immunology

## Abstract

**Objective:**

To investigate the clinical features of patients with definite vestibular migraine (dVM).

**Methods:**

A total of 91 patients with vestibular symptoms accompanied by migraines/migraine-like symptoms were enrolled and diagnosed according to the criteria of the Bárány Society and the International Headache Society. Baseline data were collected. Videonystagmography and immune-related laboratory tests were performed.

**Results:**

Among the 91 patients, 62 (68.1%) had dVM (11 men, 51 women). Among dVM patients, migraine occurred earlier than vestibular symptoms in 42 (67.7%) patients. Spontaneous vertigo occurred in 41 (66.1%) patients. Induced vertigo occurred in 21 (33.9%) patients. Motion sickness occurred in 33 (53.2%) patients. Central oculomotor dysfunction was observed in 11 (17.7%) patients. Caloric test revealed unilateral horizontal semicircular canal dysfunction in 12 (19.4%) patients. Severe intolerance during the test occurred in 44 (71.0%) patients with dVM. 12 (19.4%) patients showed abnormal immune-related indicators.

**Conclusion:**

dVM is more common in women. The onset of migraine precedes that of vestibular symptoms, which are often accompanied by photophobia and phonophobia. Motion sickness and severe caloric test intolerance can contribute to the diagnosis of dVM. Immunological indicators are abnormal in some patients with dVM, dVM may be secondary or an accompanying disorder, but the causal relationship needs further investigation.

**Level of evidence:**

IV.

## Highlights

• Definite Vestibular Migraine (dVM) is more common in women.
• The onset of migraine precedes that of vestibular symptoms, which are often accompanied by photophobia and phonophobia.
• Most patients develop motion sickness and cannot tolerate the caloric test, which can contribute to dVM diagnosis.
• Motion sickness and severe caloric test intolerance can contribute to the diagnosis of dVM.
• Immunological indicators are abnormal in some patients with dVM.

## Introduction

Both vertigo and migraine are common clinical symptoms in neurology, and their lifetime prevalence in the general population is 7% and 16%, respectively.^1^, ^2^ From the end of the 19th century, researchers have studied the relationship between vertigo and migraine.^3^ Patients with recurrent vertigo accompanied by migraine have been diagnosed with migraine-related vertigo/dizziness,^4^, ^5^ migraine-associated vertigo,^6^ and migraine-related vestibular disease.^7^ In 1999, Dieterich and Brandt^7^ first advocated the use of the term Vestibular Migraine (VM) as a diagnosis for such patients.

VM is one of the most common paroxysmal vestibular diseases, with an incidence of more than 1% in the population. The International Bárány Society and the International Headache Society jointly developed the diagnostic criteria for VM in 2012. The diagnostic criteria were published in Disorders, 3rd edition (beta version) (ICHD-3β) in 2013^8^ and in the new version, ICHD-3, in 2018.^9^ In clinical practice, the diagnostic criteria for VM mainly rely on symptoms (vestibular symptoms, migraine/migraine features) and not on evidence from objective evaluation. Therefore, the present study aimed to analyze the types of vestibular symptoms in patients with dVM diagnosed according to the criteria jointly developed by the Bárány Society and the International Headache Society, identify medical history and immune-related indicators that contribute to dVM by using multidimensional objective evaluation based on clinical characteristics, vestibular-related examinations, and immune-related laboratory tests. We hope that our study can help clinicians make early diagnosis of dVM and contribute to better patient management.

## Materials and methods

This study was approved by the institutional Research Ethics Committee (approval nº 20180314-ST-04). Written informed consent was obtained from each participant prior to recruitment.

### Subjects

A total of 91 patients with vestibular symptoms accompanied by migraine/migraine features who received treatment in the Department of Neurology, Aerospace Center Hospital, between 2018 and 2020, were included. Inclusion criteria were: (1) Age ≥ 18 years; (2) Vestibular symptoms, history of migraine; (3) Migraine defined as fulfilling items 1.1 and 1.2 in ICHD-3β;^8^ (4) Vestibular symptoms accompanied by migraine features (headache, photophobia, phonophobia, visual aura).^10^ Exclusion criteria were: (1) Presence of a new cerebral infarct; (2) Acute or chronic serious medical disease; (3) Serious ophthalmologic, otologic or cervical vertebral disease; (4) Mental disorders, anxiety or depression; (5) Vestibular symptoms caused by other known diseases (such as benign paroxysmal positional vertigo and Meniere’s disease); (6) Nervous system disease confirmed by CT and/or MRI examinations; (7) Incomplete baseline data. dVM was diagnosed according to the criteria jointly formulated by the Bárány Society and the International Headache Society.

### Clinical data

Clinical data of all patients, including demographic characteristics, clinical symptoms, past history, family history, videonystagmography, and immune-related laboratory tests were collected.

### Clinical symptoms

The following core symptoms were evaluated: vestibular symptoms (age of onset, duration, number, frequency, and cause of attacks, and accompanying symptoms); history of migraine (with or without aura, attack type, duration, causes, and accompanying symptoms); accompanying migraine features (headache, photophobia, phonophobia, visual aura); relationship between vestibular symptoms and migraine.

### Videonystagmography

Spontaneous nystagmus, gaze, saccade, visual pursuit, optokinetic, headshaking, positional and caloric tests were performed. During the caloric test, the left and right external auditory canals were irrigated with cold (30 °C) and warm (44 °C) water with the patient in the supine position and their head raised by 30°. The right external auditory canal was first irrigated with warm water, followed by the left external auditory canal with warm water and the right external auditory canal with cold water, and last the left external auditory canal with cold water (i.e., Right Warm [RW], followed by Left Warm [LW] and Right Cold [RC], and last Left Cold [LC]). There was a 5-min interval between irrigations. The Slow-Phase Velocity (SPV) during irrigation was recorded, and the Canal Paresis (CP) value was calculated. A CP value of > 25% indicates reduced unilateral horizontal semicircular canal function. A sum of the SPV values of the bilateral semicircular canals of ≤12°/s suggests reduced bilateral horizontal semicircular canal function. The criteria for hyperactive responses were as follows: total peak cool response (LC + RC) of >99°/s, total peak warm response (LW + RW) of >146°/s, total peak response (LC + RC + LW + RW) of > 221°/s. Caloric test intolerance refers to the main symptoms, including obvious nausea, vomiting, numbness in hands and feet, and body stiffness. Caloric test intolerance was considered severe if its duration was >1 h.

### Immune-related laboratory tests

Immune-related laboratory tests, including Antinuclear Antibody (ANA) test, rheumatoid factor, thyroglobulin antibody test and thyroid peroxidase antibody test, were performed. ANA test was performed using an antinuclear antibody spectrum detection kit (Diagnostic Kit for ANA Profile, LIA) to detect IgG antibodies towards nucleosome, dsDNA, histone, SmD1, PCNA, P0, SARO60 kD, SSA/RoS2D, SSB/La, CenpB, Scl70, UI-snRNP, AMA-M2, Jo-l, PM-Scl, Mi-2 and Ku in patient serum. Rheumatoid factor in patient serum was detected by immune turbidimetric assay using a rheumatoid factor detection kit. Thyroglobulin and thyroid peroxidase antibodies were detected by two-step sandwich chemiluminescence immunoassay using thyroglobulin antibody (anti-Tg) detection kit and anti-thyroid peroxidase antibody (anti-TPO) detection kit, respectively.

### Statistical analysis

Statistical analysis was performed using SPSS 22.0 software. All measurement data were expressed as the mean ± SD. The independent sample *t*-test was used to compare the means of normally distributed data between groups. Numeric data were expressed as percentage. The chi-square test was used to compare count data between groups. Yates correction for continuity or the Fisher exact test was used when necessary. All data were analyzed with a two-tailed test, and *p* < 0.05 was considered statistically significant.

## Results

### General data of included patients

A total of 91 patients with vestibular symptoms accompanied by migraine/migraine features were included in this study, there were 16 (17.6%) men and 75 (82.4%) women. Among these patients, 62 (68.1%) had Definite Vestibular Migraine (dVM) and 29 (31.9%) had probable VM (pVM).

### Clinical features of patients with dVM

Among 62 (68.1%) patients with dVM, there were 11 (17.7%) men and 51 (82.3%) women, with a male-to-female ratio of 1:4.6. Mean age at diagnosis was 46.5 ± 13.2 years (range: 23–69 years).

The age of onset of migraine and vestibular symptoms was 32.4 ± 13.1 years and 38.4 ± 15.0 years, respectively, in patients with dVM. The onset of migraine preceded the onset of vestibular symptoms in 42 (67.7%) patients. The onset of vestibular symptoms occurred earlier than the onset of migraine in 8 (12.9%) patients. Migraine and vestibular symptoms occurred simultaneously in 12 (19.4%) patients.

Thirty-four (54.8%) patients had dVM induced by precipitating factors, including sleep disorder (n = 17, 27.4%), fatigue (n = 16, 25.8%), stress (n = 6, 9.7%), menstruation (n = 4, 6.5%), emotional stimulation (n = 3, 4.8%), and seasonal changes (n = 7, 11.3%).

33 (53.2%) patients with dVM had a past history of motion sickness, 62 (100%) patients had a past history of migraine. 24 (38.7%) patients had a family history of motion sickness, 14 (22.6%) patients had a family history of migraine (Table 1).

**Table 1:** Clinical features of patients with dVM.

Clinical features	Mean ± SD/ n (%)
Age (year)	46.5 ± 13.2
Age of onset of migraine	32.4 ± 13.1
Age of onset of vestibular symptoms	38.4 ± 15.0
Sex	
Women	51 (82.3%)
Man	11 (17.7%)
Vestibular symptoms	
Spontaneous vertigo	41 (66.1%)
Internal vertigo	26
External vertigo	15
Induced vertigo	21 (33.9%)
Head-motion induced vertigo	11
Positional vertigo	6
Visually induced vertigo	2
Postural symptoms	2
Duration of vertigo attacks	
<5 min	12 (19.4%)
5–60 min	11 (17.7%)
>1 h	39 (62.9%)
Frequency of vertigo attacks	
≥1 episode/day	6 (9.7%)
<1 episode /day, ≥1 episode/week	29 (46.8%)
<1 episode /week, ≥1 episode/month	15 (24.2%)
<1 episode /month, ≥1 episode/year	12 (19.4%)
Accompanying cochlear symptoms	22 (35.5%)
Tinnitus	16 (25.8%)
Fullness of the ear	10 (16.1%)
Hearing loss	6(9.7%)
Accompanying migraine features	
Photophobia /Phonophobia	47(75.8%)
Migraine-like headache	25(40.3%)
Visual aura	18(29.0%)
Migraine	
Without aura	44(71.0%)
With aura	18(29.0%)
Vestibular symptom and migraine onset sequencing	
Migraine preceding vestibular symptoms	42(67.7%)
Vestibular symptoms preceding migraine	8(12.9%)
Migraine and vestibular symptoms occurring simultaneously	12(19.4%)
Past history	
Motion sickness	33(53.2%)
Migraine	62(100%)
Family history	
Motion sickness	24(51.3%)
Migraine	14(22.6%)

### Vestibular/ cochlear symptoms

**Figure 1:**
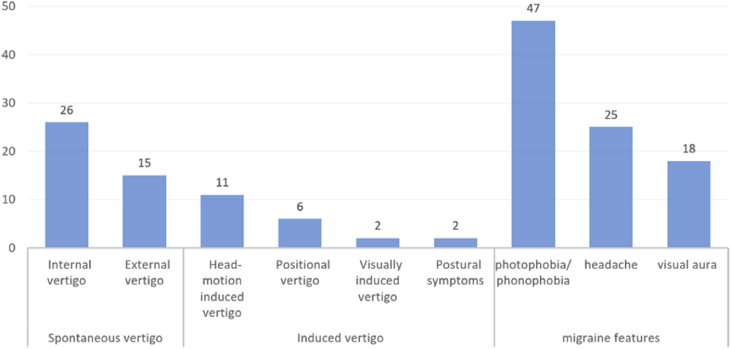
Types of vestibular symptoms.

Among patients with dVM, spontaneous vertigo occurred in 41 (66.1%) patients, including internal vertigo in 26 patients and external vertigo in 15 patients. Induced vertigo occurred in 21 (33.9%) patients, including head motion-induced vertigo in 11 patients, positional vertigo in 6 patients, visually induced vertigo in 2 patient, and postural symptoms in 2 patients (Fig. 1).

**Figure 2:**
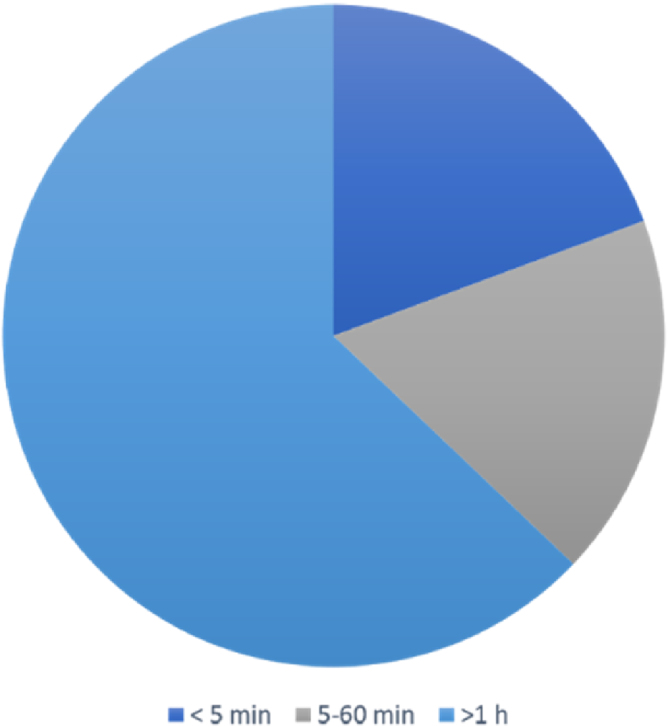
Duration of each episode of vestibular symptoms in dVM patients.

The duration of vertigo attacks lasted from seconds to days. It was less than 5 min in 12 (19.4%) patients, between 5 and 60 min in 11 (17.7%) patients, and more than 1 h in 39 (62.9%) patients (Fig. 2).

**Figure 3:**
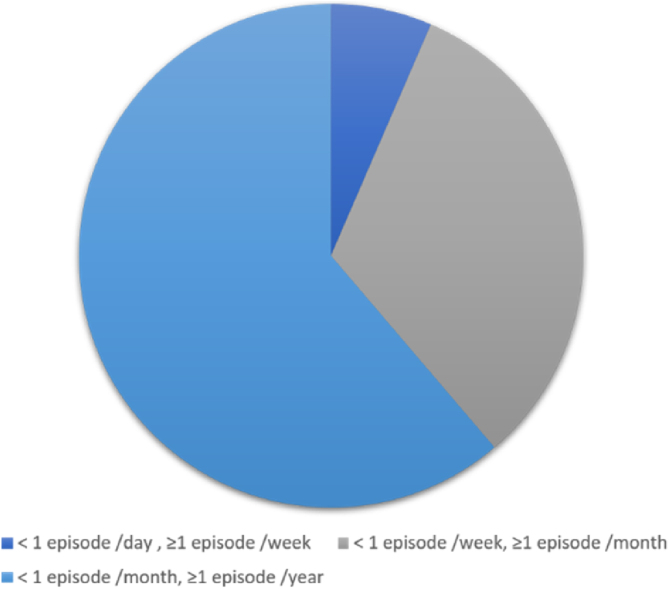
Number/frequency of episodes of vestibular symptoms.

An average of 1–6 episodes of vestibular symptoms per week occurred in 4 (6.5%) patients, an average of 1–3 episodes of vestibular symptoms per month occurred in 20 (32.3%) patients, and an average of 1–11 episodes of vestibular symptoms per year occurred in 38 (61.3%) patients (Fig. 3).

Some dVM patients were accompanied by cochlear symptoms (n = 22, 35.5%), including tinnitus (n = 16, 25.8%), fullness of the ear (n = 10, 16.1%) and hearing loss (n = 6, 9.7%) (Table 1).

### Migraine/migraine features

Vestibular symptoms were accompanied by photophobia/ phonophobia (n = 47, 75.8%), headache (n = 25, 40.3%) and visual aura (n = 18, 29.0%). And migraine without aura was found in 44 (71.0%) patients (Table 1, Fig. 1).

### Videonystagmography

All patients had no spontaneous nystagmus, gazing-induced nystagmus, or abnormal saccade. Central oculomotor dysfunction occurred in 11 (17.1%) patients, all these 11 patients had type III pursuit performance, and optokinetic test revealed bilateral weak optokinetic response in 3 patients. Static positional nystagmus occurred in 15 (24.2%) patients, and head-shaking nystagmus occurred in 19 (30.6%) patients (Table 2).

**Table 2:** Videonystagmography findings of patients with definite vestibular migraine.

	n (%)
Spontaneous nystagmus	0
Gaze test	0
Saccade test	0
Smooth pursuit test	
Type I	9 (14.5%)
Type II	42 (67.7%)
Type III	11 (17.1%)
Type IV	0
Bilateral optokinetic response weakened	3 (4.8%)
Positional nystagmus	15 (24.2%)
Head-shaking nystagmus	19 (30.6%)
Caloric test	
Unilateral semicircular canal paresis	12 (19.4%)
Hyperactive response	18 (29.0%)
Caloric test intolerance	44 (71%)

Caloric test revealed CP of >25% in 12 (19.4%) patients. Hyperactive response was found in 18 (29.0%) patients. Severe caloric test intolerance was found in 44 (71%) patients (Table 2).

### Immunological evaluation

Twelve (19.4%) patients had abnormal immune-related indicators. Among these, serum anti-thyroid antibodies were positive in 8 (including TGAb/TPOAb) patients, rheumatoid factor was positive in 2 patients, C3/C4 was positive in 1 patient, and antinuclear antibody was positive in 1 patient.

## Discussion

There is evidence that the peak incidence of dVM appears in patients at 30–60 years, and is more common in women,^7^, ^11^, ^12^ with a male-to-female ratio of 1:5. In our study, the male-to-female ratio was 1:4.6, which is consistent with previous studies. Women may be more likely to develop dVM because of changes in female hormone levels.^13^ Oh et al.^14^ found that a subgroup of patients with dVM had autosomal dominant inheritance, and the penetrance rate was lower in men than in women, with a correspondingly lower incidence in men than in women.

The onset of migraine often precedes the onset of vestibular symptoms. In our study, the onset of migraine occurred an average of 6 years earlier than vestibular symptoms in patients with dVM, and the onset of migraine preceded vestibular symptoms in 42 (67.7%) patients. Thakar et al.^15^ and Zhang et al.^16^ respectively reported that the onset of migraine occurred 8.4 and 6 years earlier than vestibular symptoms in their studied populations. The diagnosis of vestibular symptoms is the core of VM diagnosis. In clinical practice, there is great heterogeneity of vestibular symptoms in patients with VM. Neuhauser et al.^3^ showed that 67% patients with dVM had spontaneous vertigo, and 24% had positional vertigo. Cho et al.^17^ reported that head-motion vertigo with nausea is the most common symptom of dVM. Teggi et al.^18^ found that internal vertigo (73%) is more common than external vertigo (25%) in patients with dVM. Cohen et al.^19^ showed that unsteadiness (n = 134, 91%) was the most common vestibular symptom in patients with dVM. In our study, spontaneous vertigo (n = 41, 66.1%) was the most common vestibular symptom, and internal vertigo is more common than external vertigo. These findings are consistent with the results of previous studies. The heterogeneities in vestibular symptoms may be partly related to the patients’ subjective symptom description. The occurrence of these vestibular symptoms is speculated to be related to increased sensitivity of the vestibular system.^20^, ^21^

In clinical practice, there is great heterogeneity in the duration of vestibular symptoms in patients with dVM.^10^ In our study, the duration of vestibular symptoms was more than 1 h in most patients with dVM (n = 39, 62.9%), which is similar to the results reported in a previous study.^22^ Teggi et al.^18^ reported that 55.2% patients had vertigo attacks lasting more than 1 h. Ren et al.^23^ showed that vertigo attacks generally lasted several hours (37%). There is evidence that the frequency of vestibular symptoms varies greatly. In a study involving 85 patients with Dvm^24^ reported that 35 (45.5%) patients had episodes of vestibular symptoms once per day to once per week. In our study, most patients with dVM had episodes of vestibular symptoms once per year (61.3%). This variability may be caused by differences in the definition of a single episode among doctors. Morganti et al.^24^ defined the attack frequency as the number of attacks in the recent onset cycle/the onset cycle, and the frequency in this study was defined as the total number of attacks/ the total course of disease.

In our study, migraine without aura was most common in patients with dVM (n = 44, 71.0%). A previous study^16^ showed that 53 (79%) patients had migraine without aura, 8 (12%) had migraine with aura, and 6 (9%) had chronic migraine. Cho et al.^17^ reported that in 65 patients with dVM, the number of patients with migraine without aura was much higher than the number of patients with migraine with aura (66.2% vs. 4.6%).

In our study, among the accompanying migraine features, photophobia/phonophobia (n = 47, 75.8%) was the most common migraine features, followed by migraine-like headache (n = 25, 40.3%) and vision aura (n = 18, 29.0%) during dVM attacks. These results are consistent with those reported by Zhang^16^ and Xu.^25^

There is evidence that vestibular symptoms and migraine features do not occur in a specific order at each episode of VM.^7^, ^12^ Akdal et al.^26^ found that among patients with migraine with vertigo, 40% did not develop migraine accompanied by vertigo, while 17% developed migraine accompanied by vertigo. Zhang et al.^16^ showed that migraine-like headache was accompanied by vertigo in 23 (34%) patients. In our study, vestibular symptoms accompanied by migraine features was found in 25 (40.3%) patients. This may be because we investigated the order in which the vestibular symptoms and migraine features occurred during VM attack, and migraine features included not only headache, but also photophobia, phonophobia and visual aura.

We also found that migraine in patients with VM display family aggregation, which is similar to previous studies.^27^, ^28^, ^29^, ^30^ Langhagen et al.^31^ found that 65.2% patients had a family history of migraine. A study conducted by Teggi et al.^18^ involved 252 patients with dVM, and found that 177 (70.2%) patients had a family history of migraine.

In our study, approximately 53.2% of patients with dVM had motion sickness. Akdal et al.^26^ reported that motion sickness was more common in the migraine with vertigo group than in the migraine group (75% vs. 50%). Langhagen et al.^31^ reported that 51% patients with dVM had a history of motion sickness. Thus, a history of motion sickness can contribute to the diagnosis of dVM in the early stage.

Several studies have shown that patients with dVM exhibit central oculomotor dysfunction, but the incidence differs greatly among studies, ranging from 8.6% to 65.6%.^7^, ^32^, ^33^ Dieterich and Brandt^7^ showed that 59 (65.6%) patients with VM developed central oculomotor dysfunction, 43 (48%) had abnormal saccadic pursuit, 24 (27.0%) had gaze-evoked nystagmus, 10 (11%) had positional nystagmus, 10 (11.0%) had spontaneous nystagmus, and 1 (1.1%) had oculomotor nuclear palsy. A study conducted by Radtke et al.^34^ involving 60 patients with VM reported that 9 (15%) patients had central oculomotor dysfunction, including 7 (12%) of positional nystagmus, 1 (2%) of spontaneous nystagmus, and 1 (2%) of head-shaking nystagmus. They also reported a reduction in the unilateral vestibulo-ocular reflex gain in 1 (2%) patient, but no abnormalities in smooth pursuit eye movement or gaze test were found. In our study, central oculomotor dysfunction occurred in 11 (17.1%) patients, static positional nystagmus occurred in 15 (24.2%) patients, and head-shaking nystagmus occurred in 19 (30.6%) patients. The great variability in central oculomotor dysfunction among studies may be due to that investigator included different testing items of central oculomotor dysfunction. Dieterich and Brandt^7^ assessed central oculomotor dysfunction with not only the gaze test, saccadic test, smooth pursuit, spontaneous nystagmus, positional nystagmus, but also oculomotor nerve palsy. Radtke et al.^34^ also included head-shaking nystagmus and unilaterally reduced vestibulo-ocular reflex gain in video head-impulse test.

In clinical practice, patients with VM often have peripheral damage, including hearing or peripheral vestibular function reduction. Here, we found that 35.5% patients had cochlear symptoms, and 16 (25.8%) patients had tinnitus, the results are consistent with previous studies.^18^, ^35^, ^36^, ^37^ Patients with VM may have transient or persistent hearing or vestibular dysfunction, which often returns to normal after a period.^38^ This symptom can be explained by the trigeminovascular theory, this theory indicates that during migraine attacks in VM patients, the trigeminal nerve is stimulated, and substance P, neurokinin A, nitric oxide and calcitonin gene-related peptide are released, resulting in increased labyrinthine blood flow, changes in vascular permeability, and inner ear dysfunction.

In our study, most patients with dVM experienced caloric test intolerance. Previous studies showed that patients with dVM are sensitive to external auditory canal irrigation with cold and warm water.^39^, ^40^, ^41^, ^42^, ^43^ Patients with dVM developed severe intolerance after external auditory canal irrigation with cold and warm water, including obvious nausea, vomiting, palpitations, numbness of the hands and feet, and even body stiffness. In some patients, those symptoms persisted for several hours after the caloric test was completed. The mechanism underlying the hyperactive response may involve an increase in the time constant of the vestibular ocular reflex caused by heightened sensitivity of the vestibular system.^42^

It is noteworthy that in our study, serum anti-thyroid antibodies (including TGAb and TPOAb) were positive in 8 (12.9%) patients. TGAb and TPOAb are specific autoantibodies associated with autoimmune thyroid disease, indicating thyroid dysfunction. Wen et al.^44^ performed a large-sample-size study in a healthy population, and found that TGAb and TPOAb were positive in 8.97% and 9.58% of individuals, respectively. Thus, patients with dVM are positive for TGAb and TPOAb at higher rates than the healthy population, suggesting that dVM may be a secondary/concomitant condition.

Objective evaluation of dVM based on clinical characteristics, vestibular-related examinations, and immune-related laboratory tests offers opportunities for early diagnosis of dVM, however, the sample size in this study is small, and the results are mainly descriptive, further studies with larger sample sizes are needed to confirm our findings.

## Conclusion

dVM is more common in women, the onset of migraine precedes that of vestibular symptoms, and vestibular symptoms are often accompanied by photophobia and phonophobia. Most patients develop motion sickness and cannot tolerate the caloric test, which can contribute to dVM diagnosis. Some patients with dVM have abnormal immunological indexes, indicating that the pathogenesis of dVM might involve autoimmune mechanisms.

## Data availability

Data will be made available on request.

## Funding

The study was supported by Medical and Health Research Project of China Aerospace Science and Industry Corp (2017-LCYL-006).

## Conflicts of interest

The authors declare no conflicts of interest.
